# A Novel Smart Chair System for Posture Classification and Invisible ECG Monitoring

**DOI:** 10.3390/s23020719

**Published:** 2023-01-08

**Authors:** Leonor Pereira, Hugo Plácido da Silva

**Affiliations:** 1 IST—Instituto Superior Técnico, 1049-001 Lisboa, Portugal; 2 IT—Instituto de Telecomunicações, 1049-001 Lisboa, Portugal

**Keywords:** invisibles, sensory chair, posture classification, single-lead ECG, occupational health

## Abstract

In recent years, employment in sedentary occupations has continuously risen. Office workers are more prone to prolonged static sitting, spending 65–80% of work hours sitting, increasing risks for multiple health problems, including cardiovascular diseases and musculoskeletal disorders. These adverse health effects lead to decreased productivity, increased absenteeism and health care costs. However, lack of regulation targeting these issues has oftentimes left them unattended. This article proposes a smart chair system, with posture and electrocardiography (ECG) monitoring modules, using an “invisible” sensing approach, to optimize working conditions, without hindering everyday tasks. For posture classification, machine learning models were trained and tested with datasets composed by center of mass coordinates in the seat plane, computed from the weight measured by load cells fixed under the seat. Models were trained and evaluated in the classification of five and seven sitting positions, achieving high accuracy results for all five-class models (>97.4%), and good results for some seven-class models, particularly the best performing k-NN model (87.5%). For ECG monitoring, signals were acquired at the armrests covered with conductive nappa, connected to a single-lead sensor. Following signal filtering and segmentation, several outlier detection methods were applied to remove extremely noisy segments with mislabeled R-peaks, but only DBSCAN showed satisfactory results for the ECG segmentation performance (88.21%) and accuracy (90.50%).

## 1. Introduction

Today, most high-income countries’ economy has transitioned from the production of goods to the provision of services [1]. This transition has resulted in an increasing number of people employed in sedentary occupations, in which work is primarily carried out while sitting down [2]. In fact, a 2017 Eurostat report found that 39% of employees in the EU spent the majority of work hours seated(https://ec.europa.eu/eurostat/web/products-eurostat-news/-/DDN-20190305-1, accessed on 10 March 2022).

According to the European Agency for Safety and Health at Work, office workers are by far the most at risk of prolonged static sitting [3], with research showing these individuals can spend between 65% to more than 80% of work hours sitting [4], where prolonged static sitting bouts account for 55% of total work hours [5]. This places office workers at an increased risk for: Cardiovascular Diseases (CVDs) related to sedentary behavior [6]; and Musculoskeletal Disorders (MSDs) in the back, neck, shoulders, and limbs, particularly when awkward/poor postures are adopted [7,8].

In the EU, up to 100 million citizens suffer from some type of MSD, of which over 40% are work-related, making these disorders the cause for almost 50% of absenteeism and 60% of permanent work incapacity, leading to direct and indirect costs estimated at €240k million annually [9]. CVDs, on the other hand, are currently the leading cause of death worldwide, with the number of deaths expected to rise to 23 million by 2030, compared to 17 million in 2017 [10], and the total global cost of treatment expected to increase from USD 863 k million in 2010 to approximately USD 1 billion by 2030 [11].

The aforementioned effects, associated with prolonged static sitting in the workplace, ultimately represent a serious economic and social issue for both employers and overall society, due to increased absenteeism, health care, disability, and worker’s compensation costs, as well as lost productivity.

In light of these issues, there has been a growing interest in the development of systems capable of continuously monitoring either employees’ sitting behavior and posture, or cardiovascular health, but, as of now, no system proposed in the literature has integrated both of these monitoring modules. Sitting Posture Monitoring Systems (SPMSs) can be roughly divided into three main categories: computer vision based systems, wearable sensor systems and sensory chairs [12]. Even within each of these categories, system design and sitting posture classification approaches vary widely, but sensory chair-based SPMSs are by far the most ubiquitous in terms of sensor integration, user comfort and practicality.

In addition, taking into account that CVDs are a leading cause of death worldwide in developing and developed countries alike, early detection is key to prevent severe and disabling events. The Electrocardiogram (ECG) is a first-line clinical diagnosis exam for cardiovascular health assessment, in particular for the diagnosis of cardiac arrythmias, typically acquired through self-adhesive pre-gelled electrodes placed on the body. However, existing approaches are either extremely impractical in the context of office work or require collaboration of the users (e.g., touching a smartwatch worn by the user to enable measurement). Rather than requiring sensors to be placed explicitly on the user’s body, “invisible” systems, which incorporate sensors in everyday use objects or in the the environment around the user, are much better suited, especially for the early diagnosis and treatment of CVDs [13], and sensory chair-based SPMSs have the potential to easily incorporate sensors with the capacity for “invisible” ECG signal acquisition [14,15].

As such, this article proposes a smart chair system that, through an “invisible” sensing approach, can monitor conditions found to impact workers’ productivity, well-being and health, such as the user’s sitting behavior, posture, and ECG signal, without interfering in their daily activities. Thus, the focus of this work was to build upon the state-of-the-art by developing a SPMS and ECG monitoring module, which could be easily incorporated into an office chair (using low-cost hardware to facilitate large scale deployment), and to validate and evaluate the performance of these modules using data collected from them, including novel datasets for training and testing machine learning models for posture classification.

The remainder of the work is organized as follows. Section 2 describes previous work found in literature and addressing related topics. Section 3 presents our proposed approach to sensory chair-based SPMS. Section 4 summarizes the experimental evaluation results, and Section 5 discusses the performance of the proposed approach with other systems in the literature. Finally, Section 6 highlights the main conclusions and future work perspectives.

## 2. State-of-the-Art

SPMSs can be divided into three main categories, according to how relevant information to classify sitting posture is gathered. Each category has its advantages and drawbacks, but interest in the development of sensory chair-based SPMSs has grown significantly more comparatively to the other two. This growing interest can be explained by these systems’ “invisible” sensing approach, wherein sensors are ubiquitously integrated into the chair, without affecting the person’s comfort or work, enabling near-continuous and highly pervasive posture monitoring, while simultaneously avoiding the limitations of wearable systems (e.g., discomfort, users needing to remember wearing them, increased risk of damage to the sensors due to wear or external factors, abandonment issues) and computer vision based systems (e.g., image quality and camera position effect on performance, privacy concerns, incorrect detection when multiple people are in view).

The concept of sensory chairs to monitor sitting posture was first introduced in 2001 by Tan et al. [16], who integrated sensor arrays on a chair’s seat and backrest to gather pressure distribution patterns, used to classify posture. Over the years, numerous sensory chair-based SPMSs have been proposed in the literature, using a variety of sensors and classification methods with a myriad of features, extracted from the sensor data, to recognize several types of sitting positions.

**Figure 1:**
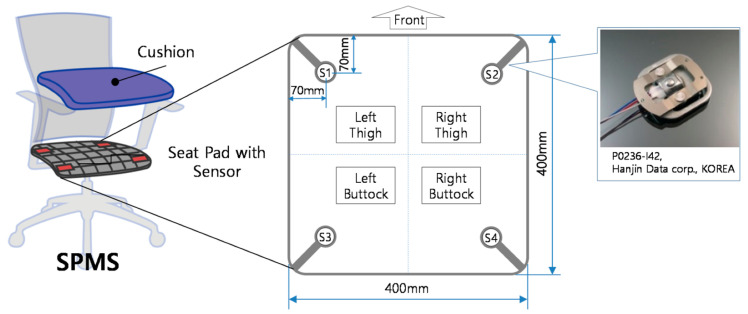
Arrangement and structure of the load cells in the SPMS. Retrieved from [17].

Roh et al. (2018) [17] developed a SPMS using four beam-type load cells integrated into the seat frame, as shown in Figure 1, to classify six postures using a feature vector composed by the ratio of total weight measured by the sensors to body weight, and of weight distribution in the medial-lateral and the anterior-posterior directions. Using a Support Vector Machine (SVM) with the radial basis function kernel, an average accuracy rate of 97.20% was obtained in the classification of postures from nine subjects.

Huang et al. (2017) [18] also used pressure sensors in their SPMS, but in the form of a 52 × 44 piezo-resistive sensor array, to measure the pressure patterns in the chair seat of eight sitting positions, used as input for an Artificial Neural Network (ANN) classifier. The average accuracy obtained was 92.2%, lower than the accuracy obtained by Roh et al. (2018) [17]. One may argue that, by evaluating more sitting positions, the system’s posture monitoring capacity increases by being prepared to distinguish between more postures, which ultimately is preferable for the promotion of dynamic sitting, i.e., the periodic alteration of sitting position. This is the currently recommended guideline by OSH agencies, in detriment of maintaining a posture that is ‘as upright as possible’ at all times, which is no longer considered ideal [3]. However, in this case, the likelihood of having positions which the SPMS might struggle to differentiate between, e.g. due to their similarity in the feature space, also increases. This trade-off is an important factor to take into consideration in the development of a SPMS.

The SPMSs mentioned above all used pressure sensors in their approach, but there are other systems that employ instead sensors capable of measuring the chair seat and backrest tilt to classify sitting posture. Otoda et al. (2018) [19] uses a total of eight accelerometers (six on the seat and two on the backrest) to measure the inclination angle variation in the *X*, *Y* and *Z* axis, which forms the feature vector used to train a Random Forest model to classify 19 postures. However, the average accuracy rate, evaluated in data collected from 20 subjects, was only 80.1%, potentially due to the higher number of postures evaluated. Additionally, using this type of sensors is only feasible, if the chair is more prone to tilting or deformable with movement, such as chairs with mesh instead of foam cushions.

There are also mixed sensor SPMSs, as is the case of the system proposed by Jeong et al. (2021) [20], which combines six pressure sensors in the seat with six infrared distance sensors in the backrest, granting it a higher capability to classify postures that only differ in terms of distance between the user’s back and the chair’s backrest (e.g., positions with trunk rotation). As such, for eleven sitting positions, this system was still able to achieve an overall accuracy rate of 92.0% using a k-Nearest Neighbor (k-NN) model, but this does come at the expense of higher system complexity and cost.

A number of SPMSs proposed in the literature (including those that have already been mentioned above) have been summarized in Table 1 and Table 2, according to the type and location of sensors used, the number of sitting positions considered, and the classification method employed. By analyzing these tables, it can be concluded that the classification accuracy achieved by sensory chair-based SPMSs varies widely, depending on the overall approach taken, which includes the sensor type and location, the number of sitting positions evaluated, and the classification methods used. Nevertheless, there is a clear trend towards the use of pressure sensors and machine learning models for posture classification.

Additionally, more complex and high-precision SPMSs that use pressure sensor mats in their approach are oftentimes able to maintain high accuracy scores when trained to classify a considerable amount of sitting positions, some of them very similar to each other, as is the case in the work developed by Huang et al. (2017) [18]. However, using these sensors can hinder large scale deployment of the SPMS, since pressure sensor mats can be very expensive (e.g., the CER2 CONFORMat^™^ sensor with 2048 sensing elements from Tekscan^™^ is commercially available for USD 3000), and may need to be replaced as a whole, if individual sensing elements in the array are damaged, which becomes more likely in the context of everyday use in an office environment. Furthermore, computing features from the high dimensional data generated by these sensors in real-time also has a high computational cost. As such, this work sought to develop a simpler SPMS, more suitable for deployment at scale, using fewer, inexpensive and more easily replaceable sensors, which could achieve similar or better accuracy scores considering more sitting positions than other SPMSs proposed in the literature.

## 3. Proposed Approach

### 3.1. System Architecture

**Figure 2:**
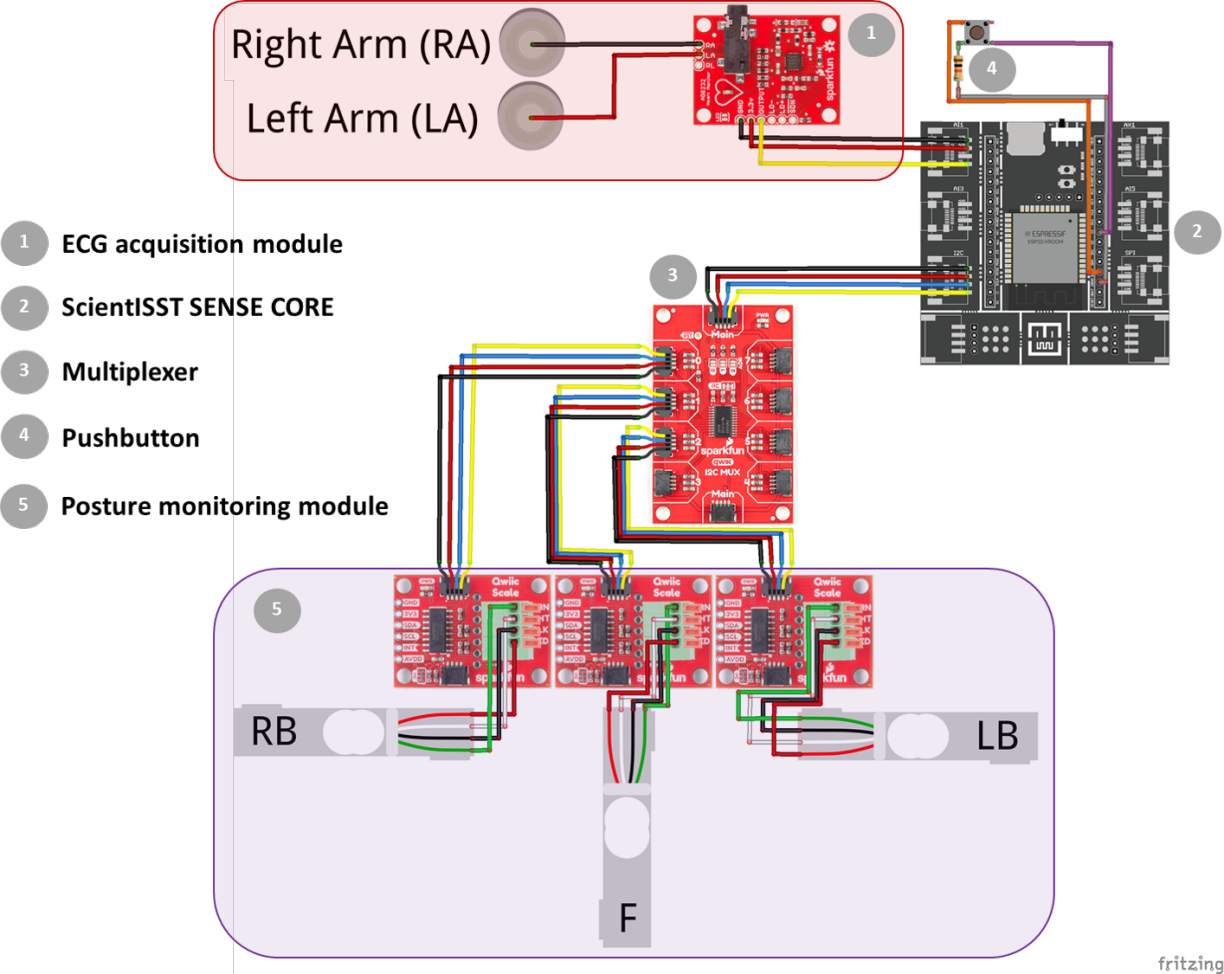
Diagram of the prototype’s system architecture and constituent hardware modules. Created with Fritzing.

In this work, a smart chair system was developed with sitting posture and ECG signal monitoring modules, which are connected to a microprocessor that handles the acquisition and transmission of the collected data, following an “invisible” sensing approach. Figure 2 depicts the diagram of this prototype’s system architecture, depicting the constituent hardware modules mentioned above, along with other supporting components.

#### 3.1.1. Posture Monitoring Module

**Figure 3:**
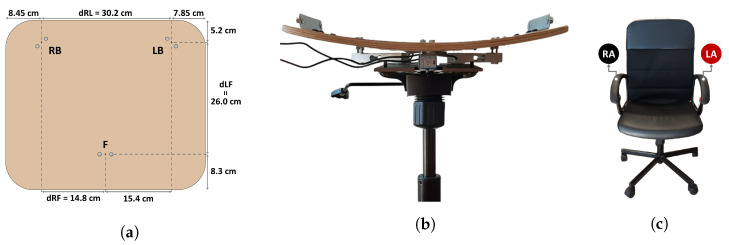
Proposed SPMS and ECG acquisition system: (**a**) diagram of the load cells’ point measurements in the chair seat; (**b**) front view of the SPMS; (**c**) position of the ECG electrodes in the chair, right arm (RA) and left arm (LA).

The SPMS component of this prototype was fabricated by integrating 3 single point load cells into the chair’s base frame (anchoring end) and seat (measurement surface) in a triangular configuration (Figure 3), to measure the weight applied at the middle point of the seat, towards the front, and at the left and right sides, towards the back. Hereafter, the load cells placed in the aforementioned points will be referred to as F, LB and RB.

These load cells, which have a maximum capacity of 50 kg each, were chosen for their high frequency response for dynamic loads and accuracy (even in cases of uneven weight distribution). The load cells are each connected to an Analog-to-Digital Converter (ADC) with a programmable gain amplifier and sampling rate (SparkFun Qwiic Scale), which, with the correct calibration, also provide weight readings. These ADCs provide a standard two-wire interface compatible with I2C protocol for a straightforward connection to the microprocessor, but, as all three have the same I2C address, a multiplexer was also incorporated into the prototype (SparkFun Qwiic Mux) to split the SCL/SDA upstream pair of the I2C bus into eight downstream channels, allowing for only one active channel at any given time, selected by the microprocessor, and avoiding address conflicts.

#### 3.1.2. Electrocardiography (ECG)

Regarding the incorporation of sensors with the capacity for “invisible” ECG signal acquisition in chairs, proposed setups in the literature acquire these signals from the back using capacitive coupled dry [15] or electro-textile [14] electrodes placed on the back and/or seat of a chair. While these setups have been shown to capture high quality ECG signals without direct conductive contact, enabling convenient long-term cardiac monitoring, they are quite complex, requiring a high-input-impedance amplifier to amplify the ECG signals acquired through clothes.

To avoid this, in our work, an “invisible” ECG signal acquisition paradigm was implemented using dry electrodes, composed of conductive nappa leather, applied to the chair’s armrests, which were previously covered with foam to facilitate grasping (Figure 3) and avoid decreased contact area between the skin and the nappa. This way, direct conductive contact between the user and electrodes becomes possible, eliminating the need for complex circuitry.

Additionally, the proposed paradigm uses only two active electrodes (ground free), an hypotheses explored by other researchers [30,31] which, despite naturally being more prone to noise, has been shown to still be capable of acquiring signals where the morphology of the ECG is maintained. This approach is advantageous comparatively to an analogous Right Leg Drive (RLD) scheme, as it also requires less components (e.g., conductive fabric, connecting cables, and assembly), and is not dependent on the positioning of the arm in the armrest.

Furthermore, the same electrode material used here has also already been implemented in a similar approach for the acquisition of ECG signals from the keyboard armrest and computer mouse [30], achieving high correlation between the signals obtained by the proposed device and a reference sensor. These previous results suggest that the conductive nappa provides an adequate electrical interface with the skin in most cases.

The conductive nappa electrodes in this prototype were connected to the SparkFun Single Lead Heart Rate Monitor, which is an integrated analog signal conditioning frontend for ECG applications, configured to amplify and filter the measured signal, limiting its bandwidth to the typical ECG monitor frequency ([0.5; 40] Hz), thereby reducing noise. In the end, the amplified and filtered ECG signal is output as raw analog data.

#### 3.1.3. Acquisition and Transmission Module

In order to control the data acquisition from the aforementioned sensor modules, the ScientISST SENSE CORE (seen in Figure 2) was incorporated into this prototype. Hereinafter, this device is referred to as SENSE.

The SENSE board includes many features that make it the ideal microprocessor for this system, including: two low-power Xtensa^®^ 32 bit LX6 microprocessor cores; six analog input ports with 12 bit resolution to connect any sensor with a compatible analog output, such as the ECG sensor; an I2C port to establish a communication line between the SENSE board, load cell ADCs, and multiplexer; support for wireless communication protocols (Wi-Fi, Bluetooth and BLE); operable with any rechargeable LiPo battery using a supply voltage above 3.3 V; two separated regulated 3.3 V outputs to independently power the analog and digital parts of the acquisition circuit; and a USB-C connector to recharge the connected battery and establish a serial communication line between the board and a PC for data transmission and reprogramming purposes.

A purpose-built C++ firmware was devised in the Arduino IDE, using pre-built libraries with the functions necessary to easily interface SENSE with the aforementioned devices. Several versions of this firmware were developed, depending on the experiments being conducted, as will be discussed next.

### 3.2. Data Collection

The prototype described in the previous section was evaluated with experimental data collected in two separate phases.

**Figure 4:**
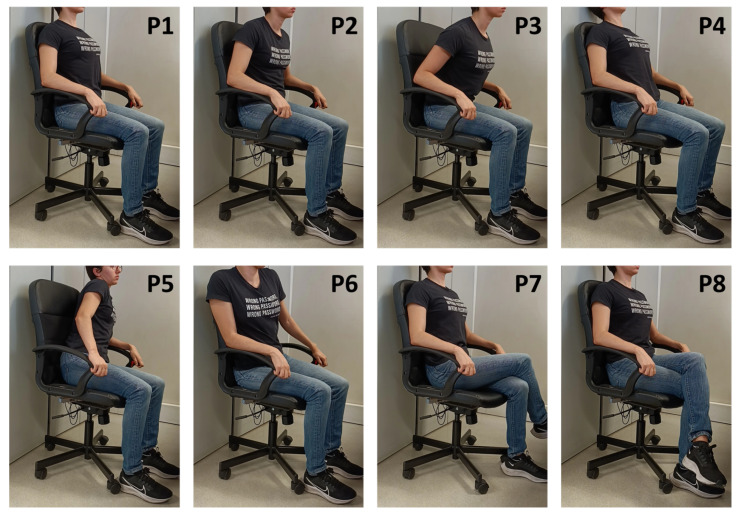
Sitting positions used in the experiments and their respective class label: (**P1**) Upright sitting; (**P2**) Slouching; (**P3**) Leaning forward; (**P4**) Leaning backward; (**P5**) Leaning left; (**P6**) Leaning right; (**P7**) Right leg crossed; and (**P8**) Left leg crossed.

In the first phase (A), 10 healthy subjects (5 Female, Age: 30.8 ± 13.1, Weight [kg]: 63.3 ± 11.0, Height [cm]: 170.8 ± 5.8, BMI: 21.6 ± 2.6) were asked to mimic a series of eight seated postures (P1 to P8, depicted in Figure 4), without leaving the chair and keeping their arms and hands in contact with the armrests, after receiving instruction on how to adopt these positions by the examiner, emptying their pockets and adjusting the chair height to ensure a 90° angle at the knee.

In this experiment, weight data from the load cells (in grams) was acquired during periods of static sitting for each evaluated posture, meaning data acquisition was only initiated after the subject was seated adequately and stably in one of the eight positions, and movement was kept to a minimum during acquisition, which lasted 60 seconds. Forty acquisitions were conducted for each subject, as this process was repeated in five separate days to assess if choice of clothing or slightly different chair height between days could impact the posture classifiers’ performance. The motivation for collecting these data was to later train several machine learning models as detailed in Section 3.3.2.

Simultaneously, in this experiment data were acquired from the proposed ECG acquisition module and a reference system, composed by a second single-lead ECG sensor connected to disposable pre-gelled electrodes, and applied to the participants’ body in an Einthoven’s Lead I configuration. With these data, the aim was to evaluate the performance of the proposed system in comparison to the reference. In this experiment, sampling rates of 320 Hz and 500 Hz, were used respectively for load cell and ECG data acquisition, the former using a resolution of 24 bits and the latter using 12 bit resolution.

In the second phase (B), the focus was to only acquire weight data from the load cells, particularly from users unfamiliar to the posture classification models trained beforehand, in order to extract the same type of features and create a test dataset with which to evaluate the performance of these models. For this purpose, 22 healthy subjects (9 Female, Age: 23.5 ± 1.9, Weight (kg): 65.3 ± 11.5, Height (cm): 172.5 ± 7.5, BMI: 21.8 ± 2.6), 15 of which did not participate in experiment A, were asked to follow the same protocol used before, but with some notable differences, namely: continuous data acquisition, even during the transition period between positions, with the start and end of static sitting periods, for each position, marked by the press of a pushbutton, incorporated into the prototype; and no specific instructions given on hand placement to ensure classification accuracy was not significantly impacted, if the user places their hands on their lap or table. In this case the sampling rate for the load cells was reduced to 20Hz.

In both phases, during acquisition, data were transmitted from the ScientISST SENSE Core using serial communication to a computer terminal (with an Intel®Core^™^ i7-8750H CPU, a base processor frequency of 2.2 GHz, and 16 GB RAM), where it was recorded and stored as CSV files to be later processed in the same terminal.

### 3.3. Data Processing

#### 3.3.1. Electrocardiography (ECG)

**Figure 5:**
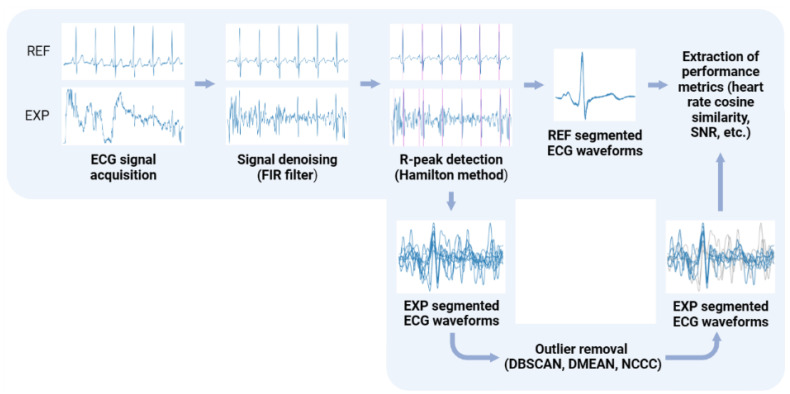
Processing pipeline for the ECG signals acquired with the reference sensor (REF) and the proposed “invisible” system (EXP). Created with BioRender.com.

The diagram depicted in Figure 5 details the processing of the acquired ECG signals in these tests, which includes the use of a digital Finite Impulse Response (FIR) filter of order 150 to preserve the signal only in the 3–45 Hz frequency range and the Hamilton method [32] to segment the ECG waveforms. Both the digital filtering and segmentation methods used were taken from the validated BioSPPy library (0.8.0) [33], and were selected based on previous works that used a similar “invisible” sensing approach with the same electrode material [30].

However, in some cases, the signal acquired with the conductive nappa was contaminated by a considerable amount of noise, most likely due to weak contact between the skin and armrests, the triboeletric effect, or high skin impedance caused by skin moisturizers [30], leading to erroneous R-peak detection. As a result, three methods for outlier detection, used previously by other research works when faced with this same problem, were applied to the segmented heartbeat waveforms to try and disregard segments resulting from mislabeled R-peaks, namely: DBSCAN [34], DMEAN [35], and NCCC [31]. The first two methods define a cluster of “normal” heartbeats based on the distance between them in their feature space using some metric (in this case, cosine distance), with DBSCAN using a fixed minimum threshold (ϵ=0.45) and a number of neighboring heartbeats to define this cluster (MinPts=10), whereas DMEAN uses adaptive thresholds (in this case, the same parameter values used in [35]). Lastly, NCCC defines a cluster based on the average normalised cross-correlation (*A*) of each heartbeat waveform, by setting the initial cluster as the 20 waveforms with highest *A* and then going through the remaining ones in descending order, until one does not meet the conditions set out in [31], with all remaining heartbeats deemed as outliers.

After these pre-processing steps, the following metrics were extracted from the signals: heart rate for segments in which two or more consecutive R-peaks were available; R-peak amplitude; SNR and cosine similarity of the experimental filtered signals compared to the reference signals, after scaling. Furthermore, to better evaluate the effect of the aforementioned outlier detection algorithms in the ECG segmentation performance, several metrics were extracted from the matching segments in the reference and experimental signals, including the performance and accuracy rate, calculated using the following equations:

(1) $$Performance\;\left(\%\right)=\frac{TP}{N_{R-peaks}^{REF}}$$

(2) $$Accuracy\;\left(\%\right)=\frac{TP}{TP+FP}$$

where TP and FP are the number of true and false positives in R-peak detection, and $N_{R-peaks}^{REF}$ is the number of R-peaks in the reference signal.

#### 3.3.2. Sitting Posture Classification

Prior to feature extraction, all of the acquired load cell signals in experiment A were run through a pre-processing pipeline, starting with the removal of any extreme outlier in the data (i.e., weight reading well above the sensor’s maximum capacity) by replacing these with the previous valid weight reading. Afterwards, load cell signals were run through a moving average filter with a one second non-overlapping window, in order to smoothen the signal and reduce jitter from small movements, while also downsampling the signal; despite these steps, the needed information is preserved.

To create the feature vectors of the dataset with which to train the posture classification models, the processed load cell data collected from the three load cells was used to compute the Center of Mass (CM) in the reference frame displayed in Figure 3, using Equations (3) and (4):

(3) $$CM_{x}=\frac{dRL\;\cdot \;LB+dRF\;\cdot \;F}{RB+LB+F}$$

(4) $$CM_{y}=\frac{dBF\;\cdot \;F}{RB+LB+F}$$

where $CM_{x}$ and $CM_{y}$ are the *x* and *y* components of the CM, RB, LB and *F* are the weight measurements from those load cells, and dRL, dRF and dBF are the distances depicted in Figure 3.

Recapping, the feature vectors used to train the posture classifiers contain the CM coordinates in the 2D seat plane, computed from the average weight readings acquired from the three load cells in a 1 second non-overlapping window. As such, in this work, the posture classification task can be thought of as dividing the 2D chair seat plane into areas assigned to a specific sitting position. For this purpose, both supervised and unsupervised machine learning models were trained with the CM dataset. The former encompassed k-NN, Nearest Centroid (NC), and SVM models, whereas the latter only included the Gaussian Mixture Model (GMM) [36]. For reasons detailed in the next section, the P4 position was excluded from the dataset, and two sets of the aforementioned models were created: one to classify the five sitting positions (P1, P2, P3, P5, P6), and the other to classify seven sitting positions (P1–P3, P5–P8).

Before training these models, stratified 5-fold Cross Validation (CV) was used to tune each models’ hyperparameters and decrease the risk of overfitting during this process, by using an exhaustive grid search approach, to search for the best CV accuracy score. The only hyperparameter that was manually selected was the number of neighbors in the k-NN classifiers, which were created with fixed values for this parameter, for reasons discussed in Section 4.3. The hyperparameter space used for each classification model in this process can be found in Appendix A. Lastly, these models were re-trained with the full training dataset using the optimal hyperparameters values found in CV, to create the final posture classifiers for implementation.

To obtain the test dataset with which to evaluate the performance of these classifiers, first the already processed load cell signals output by SENSE had to be split, to extract and label the 60 second periods of static sitting for each position, using the digital signal associated to the pushbutton (set to HIGH when the button was pressed). The resulting dataset was then used to compute performance measures for each classifier, such as precision, recall, F1-score and accuracy, in order to find the best performing model among the five-class and seven-class posture classification models. These metrics together with the overall classification accuracy were obtained using Equations (5)–(8):

(5) $$Precision\left(y,\hat{y}\right)=\frac{1}{\left|L\right|}\sum_{i\in L}\frac{\left|y_{i}\cap \hat{y_{i}}\right|}{\left|\hat{y_{i}}\right|}$$

(6) $$Recall\left(y,\hat{y}\right)=\frac{1}{\left|L\right|}\sum_{i\in L}\frac{\left|y_{i}\cap \hat{y_{i}}\right|}{\left|y_{i}\right|}$$

(7) $$F_{1}\left(y,\hat{y}\right)=\frac{1}{\left|L\right|}\sum_{i\in L}\frac{2\times P\left(y_{i},\hat{y_{i}}\right)\times R\left(y_{i},\hat{y_{i}}\right)}{P\left(y_{i},\hat{y_{i}}\right)+R\left(y_{i},\hat{y_{i}}\right)}$$

(8) $$Accuracy\left(y,\hat{y}\right)=\frac{1}{n_{samples}}\sum_{n=1}^{n_{samples}}1\left(\hat{y_{i}}=y_{i}\right)$$

where *y* and $\hat{y}$ are the sets of true and predicted (sample,label) pairs, respectively, *L* is the set of labels, $y_{i}$ and $\hat{y_{i}}$ are subsets of *y* and $\hat{y}$, respectively, with label *i*. Overall, the classifier’s precision is its ability not to label as a certain sitting position a sample that in truth does not have that label, while the recall is its ability to find all samples from a specific sitting position. The F1-score combines the previous two metrics into a single metric by taking their harmonic mean. Lastly, the classification accuracy is simply the portion of correct predictions in the test dataset.

At the end, the final classifier chosen was used to predict the sitting position during the transition periods, to analyze the model’s behavior in these periods.

## 4. Results

### 4.1. Electrocardiography Performance Evaluation

The results of the comparative analysis between the ECG signals acquired with the proposed and reference systems are summarized in Table 3.

**Figure 6:**
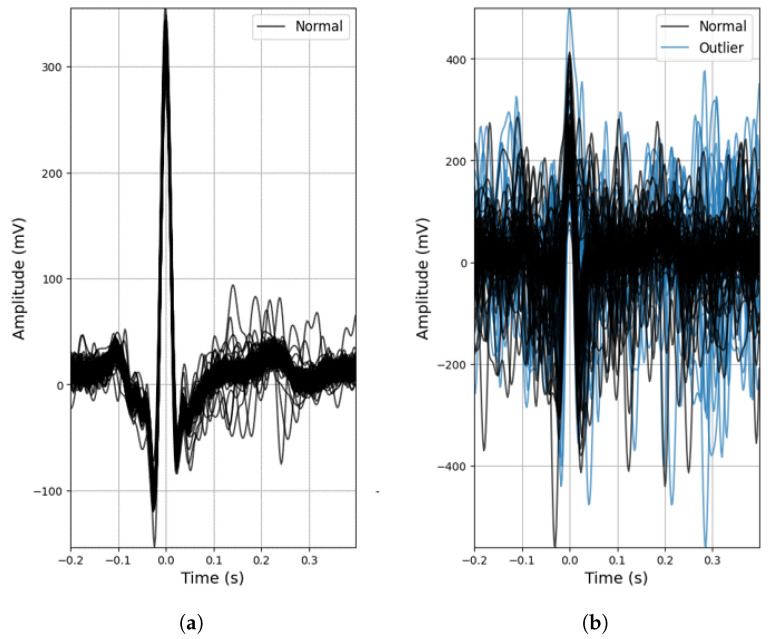
Examples of outlier detection using the DBSCAN method: (**a**) Participant 4, acquisition no. 18; (**b**) Participant 5, acquisition no. 19.

As mentioned beforehand, the conductive nappa and use of two active electrodes are more prone to noise (Figure 6b), but even so, despite the lower R-peak amplitudes (expected due to the increased impedance) and the effect of noise, for the majority of the ECG signals acquired with the conductive nappa, the general morphology of the ECG signal was maintained, as can be observed in Figure 6a, with some signals even achieving a SNR of 12.17 dB. In fact, even without any outlier detection algorithm applied to the extracted heartbeat waveforms, both the mean performance and accuracy rate of the proposed ECG system are quite high (90.83% and 85.84%, respectively), but the standard deviation of these metrics is also quite high, again indicating that some of the processed ECG signals are still contaminated by noise.

Observing the column for the heart rate metric in Table 3, without outlier removal, this noise leads leads to an overestimation of the average heart rate value and a high standard deviation, indicating the presence of false positives in R-peak detection. By disregarding these false positives using outlier removal methods, the average heart rate comes closer to the one obtained from a reference sensor with pre-gelled electrodes and the standard deviation drops significantly, with NCCC exhibiting heart rate values quite comparable to those of the reference sensor.

Overall, by applying the outlier detection algorithms, the performance of ECG segmentation decreased, meaning that these methods are removing both erroneously and correctly identified R-peaks. The extent of this decrease varied between these algorithms, depending on their sensitivity to noise. DBSCAN showed the lowest decrease (only ≈2%), reason for which it was integrated into the processing pipeline of the acquired ECG signals in the final prototype.

### 4.2. Center of Mass Evaluation

**Figure 7:**
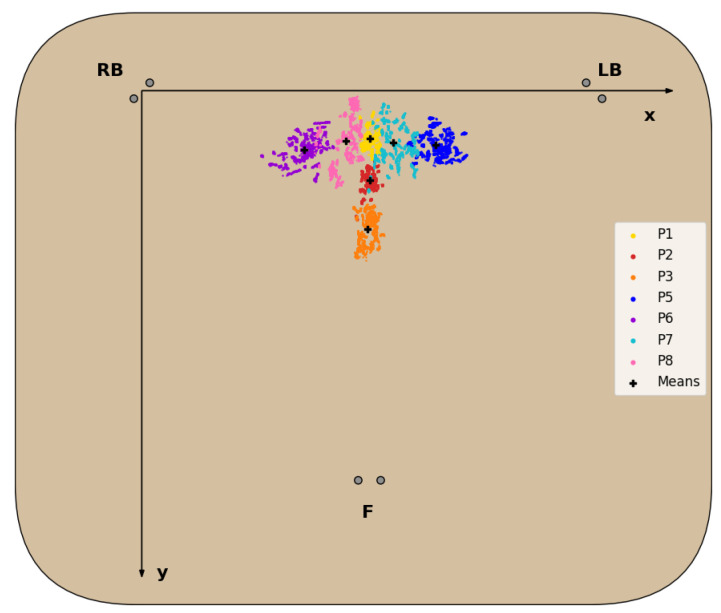
Diagram of the dataset of CM coordinates for each evaluated sitting position, except P4, and corresponding mean.

The dataset of CM coordinates, resulting from the data processing and feature extraction described in Section 3.3.2, for each sitting position (with the exception of P4 for reasons detailed next) is represented in the chair seat diagram in Figure 7, and the statistical analysis of the CM coordinates obtained is detailed in Table 4.

For all sitting positions evaluated, with the exception of P4, the corresponding CM positions obtained formed clusters in the 2D seat plane with little overlap between these clusters, as seen in Figure 7 and confirmed by the statistical data analysis in Table 4. Regarding the P4 posture, the CM positions obtained failed to form a defined cluster, due to the chair’s characteristics, which made it unable for subjects to properly and comfortably lean back, resulting in the disproportionately higher standard deviation of the $CM_{y}$, seen in Table 4, and its exclusion from the dataset.

For all other seated postures, the corresponding clusters fall on the expected areas of the chair seat. Positions involving no lateral movement and differing only in the degree of back support (P1,P2,P3) form clusters around the midpoint of the chair seat in the *X*-axis, as readings from the LB and RB sensors should be fairly similar. In the *Y*-axis, these clusters are closer to the front of the seat, the lesser the degree of back support, as more weight is shifted towards the F cell. The same logic applies to positions involving only lateral movement (P5 and P6), but in this case weight is shifted towards the left or right side, while the weight in the front remains roughly the same, resulting in the clusters most externally located in the *X*-axis. Lastly, the clusters for the P7 and P8 positions appear between the clusters of the more central and external positions mentioned above, since the leg crossing motion requires some lateral movement, but not at the same level as fully leaning to the sides.

### 4.3. Posture Classification Model Training

The optimal hyperparameters obtained from the stratified 5-fold CV process are summarized in Table 5. For five classes, the trained classifiers all display excellent validation accuracy scores (>99.5%), which was to be expected, taking into consideration the very little overlap between the clusters for these positions. On the other hand, for seven classes, the validation accuracy score of each trained classifier decreased, ranging from 88.95% to 98.72%.

Overall, the k-NN models have the highest validation accuracy scores for both five and seven classes, showing an inverse relationship with the number of neighbors used in the model. The reason behind this relationship is the formation of small “islands” in the decision regions of sitting positions where cluster overlap occurs or outliers simply exist, as in those areas the majority of neighboring samples will correspond to another sitting position, leading to correct classification in the training dataset but potential misclassification for unseen data. This behavior is a disadvantage of this model, which can be reduced by increasing the number of neighbors. The other supervised classification models (NC and SVM) generate more homogeneous decision areas, which is preferred to avoid overfitting.

Furthermore, while the validation accuracy obtained by the GMM model may seem very satisfactory for both five (99.52%) and seven classes (88.95%), this model is not suitable to classify sitting positions from unseen data when considering seven classes. This is because the GMM is a probabilistic model that assumes all data points are generated from a mixture of a finite number of Gaussian distributions with unknown parameters, so if the clusters found in the CM dataset do not approximately follow this distribution, the model will not be able to identify them from the unlabelled data, as is the case here.

### 4.4. Performance Evaluation of the Posture Classification Models

**Figure 8:**
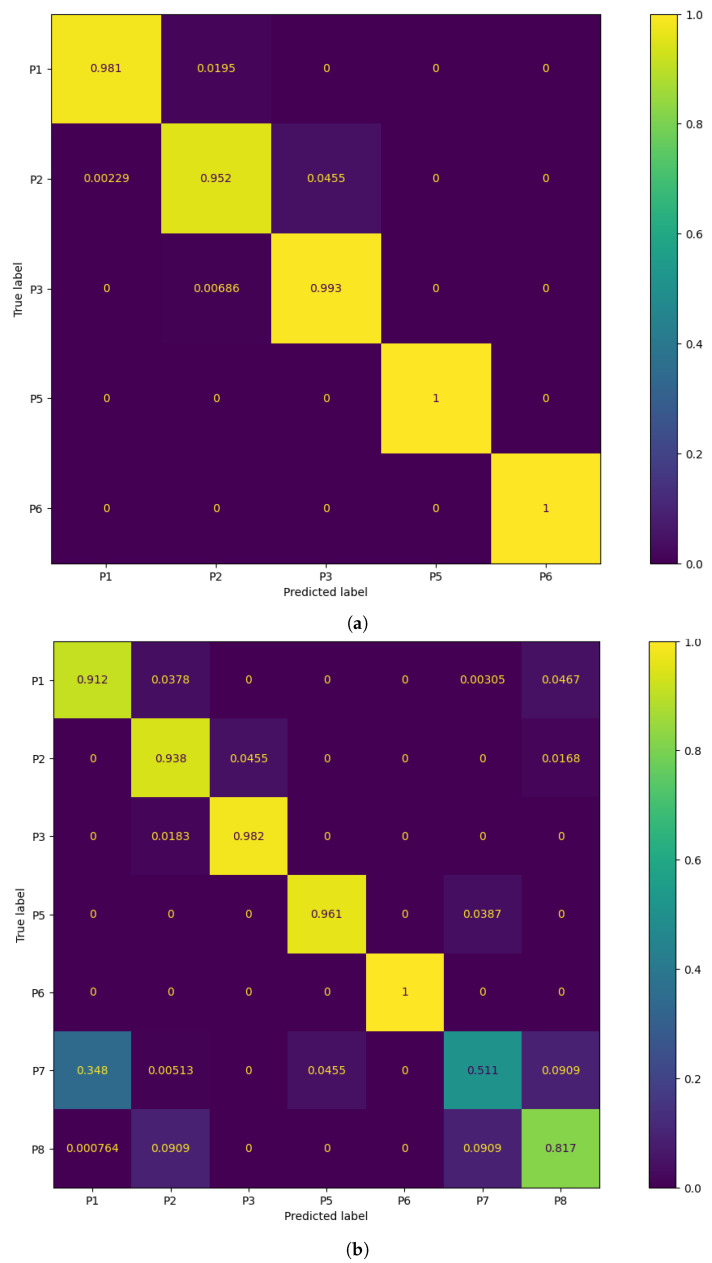
Confusion matrix of the best performing models: (**a**) 5-class k-NN using 1000 neighbors and (**b**) 7-class k-NN using 3000 neighbors.

**Figure 9:**
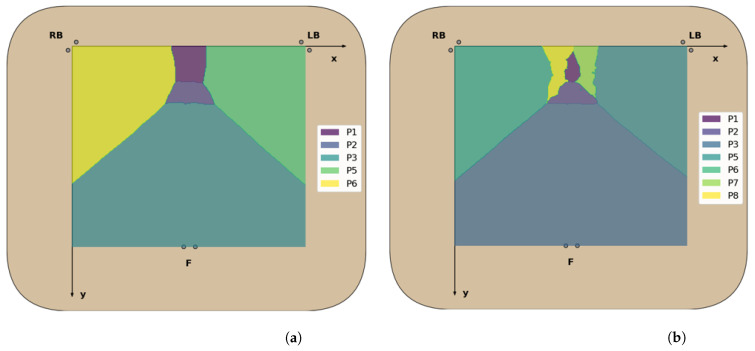
Decision boundaries of the best performing models: (**a**) 5-class k-NN with 1000 neighbors and (**b**) 7-class k-NN with 3000 neighbors.

Table 6 displays the classification performance metrics obtained using the test dataset, for each model trained. As a multilabel classification task, the precision, recall and F1-score values were obtained by averaging these metrics’ unweighted mean per label, which can be found in Appendix B.1 displayed in bar plots. Furthermore, in order to better evaluate the classification accuracy per sitting position, confusion matrices were created for each posture classifier evaluated, but only the matrices for the best performing five- and seven-class posture classifiers are displayed here (Figure 8). For a better insight into each classifier evaluated, all confusion matrices obtained can be found in Appendix B.2. Similarly, the decision boundaries in the chair seat plane of these two models are also represented here (Figure 9), but the decision boundaries of all models can be found in Appendix B.3.

Overall, for five classes, the classification models exhibit excellent values for each of the evaluated performance metrics (>97.4%), with no significant differences between the accuracy in the test dataset and the validation accuracy. This was to be expected, taking into consideration the little overlap between the clusters of CM coordinates for these positions, leading to high classification accuracy rates across all classes (Figure 8). The k-NN models had once again the best classification performance, but here the ones using a greater number of neighbors performed better than those using fewer neighbors, as was predicted in the previous section. In particular, using 1000 neighbors yielded the best performance metrics and decision boundaries (Figure 8) across all models, as using fewer did not completely eliminate the small “islands” caused by cluster overlap, and using more led to more rigid decision boundaries.

On the other hand, for the models trained to classify seven classes, there was a marked decreased in the overall accuracy comparatively to the validation accuracy, which was mostly caused by the misclassification of the P7 and P8 positions (the percentage of P7 and P8 samples labeled correctly ranged from 36.1% to 60.9% and 45.3% to 86.4%, respectively) and, to a lesser degree, of the P1 and P2 positions (77.5% to 100% and 77.3% to 93.8%, respectively). Again, this was expected due to the inherent overlap between the CM clusters of the P1/P2 and P7/P8 positions. However, overall, the classification performance metrics obtained for the supervised learning models were still satisfactory.

Furthermore, all seven-class supervised models had similar recall and precision values, leading to close F1-score values, which indicates that, on average, the classifiers’ ability to correctly label data from a specific sitting position was similar to their ability to not label data as a given sitting position which does not coincide with its true label. However, the higher standard deviation values of the precision and recall metrics compared to the five-class supervised models also indicate that the aforementioned abilities become significantly hampered for certain classes, in particular the added P7 and P8 positions, as can be seen from the bar plots displayed in Appendix B.1.

The best performing classifiers for seven sitting positions were the k-NN models, with accuracy ranging from 85.8% to 87.6%. Similar to what occurred with five positions, these models exhibited better accuracy when using a higher number of neighbors, in particular 2000 (87.6%) and 3000 (87.5%), as this leads to the definition of more homogeneous decision areas (Figure 9).

Overall, the models trained with seven sitting positions struggled in particular with the classification of P7 and P8 samples, but, while not as accurate, these models have a superior posture monitoring capacity compared to those only trained with five positions, which are consequently more limited in their implementation in a real-world environment and ability to verify dynamic sitting. As a result, the classifier chosen for the final prototype was the seven-class k-NN model with 3000 neighbors. While using 2000 neighbors with this model yielded a marginally better accuracy score, it was decided to use the model with 3000 neighbors instead, due to its higher accuracy for the P1 and P2 positions, which are the most commonly found sitting postures in the office environment among all of the evaluated positions (Figure 8).

Table 7 shows the relative frequency of the predicted label in each transition period obtained from the final posture classifier chosen, averaged across all participants. With this final model, it was verified that, during the transition periods between sitting positions, the classification was for the most part stable, with the predicted label changing from the start to the end position. Only occasionally, the predicted label changed briefly to other positions, corresponding to periods where movement occurs during the changing of position, resulting in more or less weight being applied to certain load cells.

## 5. Discussion

As was mentioned in Section 1, the focus of this work was to develop a SPMS and ECG signal acquisition module incorporated into an office chair, using an “invisible” sensing approach, and to validate and evaluate the performance of these modules. The main aim was to ensure that the system was able to record usable ECG signals and the posture classification approach taken (i.e., type and location of sensors, extracted features, classification model, evaluated sitting positions) was adequate, with accuracy surpassing or on par with other systems proposed in the literature.

For the evaluation of the proposed ECG acquisition scheme, several metrics were extracted from the signals acquired by this system and compared to those obtained by reference sensors. After the analysis of these results, we concluded that, although the proposed system is more prone to noise, the results are quite satisfactory. Even though systems that use an analogous RLD scheme are more robust to this noise, such as the one proposed by Su et al. (2021) [14], who were able to achieve a SNR between 15.02 and 25.02 dB (depending on the placement of the conductive textile electrodes on the backrest), the majority of signals acquired with the ground-free system proposed in this work still maintained the morphology of the ECG signal, achieving a SNR as high as 12.17 dB.

Additionally, without outlier removal algorithms, the average performance and accuracy rates of ECG segmentation highlight this observation, with obtained results above 90% and 85%, respectively, demonstrating this system’s feasibility. However, as expected, some of the acquired signals were contaminated with intense noise, leading to erroneous R-peak detection and overestimation of the heart rate. In the system proposed by Silva et al. (2021) [30], which used the same conductive nappa employed here (albeit wrapped around the main areas of a gaming keyboard armrest and mouse that come in contact with the hand), the average performance achieved was 99.99% and 95.83% when using two active electrodes on the keyboard armrest and one on the armrest with another on the mouse, respectively. However, the first value fell to 77.03% and 75.14% when subjects had low and high density skin moisturizers applied to their skin, respectively, leading us to conclude that these products could be a potential source of noise in the acquired signals.

Using three methods of outlier detection to the extracted heartbeat waveforms, we were able to mitigate this noise, leading to increased accuracy (ranging from around 5% to 10%) by discarding erroneously detected R-peaks in noisier segments of the signal. However, the performance also decreased (ranging from around 2% to 20%), due to the removal of correctly detected R-peaks in segments contaminated with noise. This decrease was dependent on the algorithms’ sensitivity to variance between heartbeat waveforms, with the obtained results corroborating previous studies that found DBSCAN to be the least sensitive and NCCC the most [31,35].

Nevertheless, the proposed ECG acquisition system was able to record usable signals, from which the heart rate, a metric that can be clinically relevant, can be accurately derived from. For example, this metric can be used to detect cardiac arrhythmias, especially when taking into consideration the “invisible” nature of this system, that allows for pervasive monitoring, as opposed to sporadic signal acquisition.

Additionally, this metric varies with both physical and cognitive exertion, leading to it being often treated as a surrogate for sympathetic activity and has also been found to be helpful to assess stress. Adding to this all the work conducted around the study of the heart rate variability, the heart rate has been successfully used for enhanced risk assessment in low- to intermediate-risk individuals without known coronary artery disease [37].

It is also relevant to highlight that, at least in a first deployment stage, this type of system can aid in triage of potential cardiovascular disorders rather than performing a full-featured diagnosis. This is illustrated by recent developments on the detection of atrial fibrillation (the most common type of cardiac arrhythmia globally) from single-lead ECG recordings, exhibiting excellent diagnostic accuracy, such as the algorithms used by Apple and AliveCor [38,39], as well as a morphology-based deep learning model with visualization of the ECG segments most relevant for the detection of this pathology [40]. Nevertheless, ultimately, the purpose of this ECG acquisition module is not to replace the medical professional, who is the best suited individual to diagnose cardiovascular disease, but to help their work by providing them with more data to aid in making a diagnosis.

For the SPMS proposed in this work, several posture classification models were created, trained and tested with novel datasets of CM coordinates in the 2D seat plane, in order to distinguish between five and seven sitting positions. Although the original dataset covered eight positions, the P4 position was excluded, as it was the only position to not form a cluster in the feature space. Several metrics (precision, recall, accuracy) were evaluated to find the best performing classification model, which turned out to be the k-NN model with 1000 and 3000 neighbors, for the five-class and seven-class models, respectively.

Compared with other sensory chair-based SPMSs proposed in the literature, the five-class model achieved superior classification accuracy results (98.5%), which was to be expected taking into consideration the very little overlap between the clusters of these positions. Even high-precision SPMSs did not perform this well, such as the one proposed by Kim et al. (2018) [26], who used a pressure sensor mat to generate heat map images of the body pressure distribution in the chair seat, with which to classify the same number of sitting positions using a deep learning model (CNN), achieving an accuracy of 95.3%.

On the other hand, the seven-class model exhibited decreased accuracy compared to the five-class ones, due to the overlap of P7 and P8 with the remaining positions. Even so, the classification accuracy achieved (87.5%) did not deviate significantly from other SPMSs proposed in the literature, particularly for the postures most commonly adopted in the office workplace (P1 and P2). These results are a significant contribution to the state-of-the-art, especially, when taking into consideration the overall simpler approach taken in terms of sensor and computational complexity. For instance, the accuracy achieved by the high-precision SPMS proposed by Huang et al. (2017) [18], which evaluated the same seven sitting positions considered in this work, with high-dimensional pressure maps of the chair seat, surpassed the one achieved by our system by only 5% approximately.

Furthermore, we found that even during transition periods between sitting positions, the classification with the best performing seven-class model was for the most part stable, highlighting this system’s capacity to evaluate dynamic sitting in the workplace. We also found that the execution time of this classifier was approximately 4.62±2.67 milliseconds, when evaluating CM coordinates ranging from 0 to 30.3 cm in the *X*-axis and 0 to 26.1 cm in the *Y*-axis, with a 0.1 cm step, in the chair seat reference frame. Thus, this model has been found to be adequate for real-time posture classification in a future real world deployment.

Nevertheless, in the future, we plan to also increase this system’s posture monitoring capacity by evaluating more sitting positions, such as the one used by Roh et al. (2018) [17], who achieved a high classification accuracy, using a SPMS similar to the one proposed in this work. Additionally, to try and increase the performance of posture classification, especially for the P7 and P8 positions, we will experiment adding new inexpensive sensors (e.g., infrared reflective distance sensors as proposed in [20]) and features to the dataset.

As a final note, this is one of the first smart chair systems proposed in the literature, that integrates both validated sitting posture monitoring and ECG signal acquisition modules. The overall cost of the hardware required to develop this system is only approximately 150 euros, markedly less than most systems that only integrate posture monitoring (e.g., the CER2 CONFORMat sensor with 2048 sensing elements from Tekscan^™^ used in some SPMSs is commercially available for USD 3000 [41]), facilitating its large scale deployment in the future.

## 6. Conclusions

In this work, a smart chair system was developed, with integrated sitting posture monitoring and ECG signal acquisition, following an “invisible” sensing approach.

For posture monitoring, a sensory chair-based SPMS was developed using single point load cells integrated under the seat to measure the weight applied at specific points. Posture classification was performed using the CM coordinates computed from the average weight values, in a 1 second non-overlapping window, as feature vectors, which were found to form distinct clusters for seven of the eight evaluated sitting positions. Two datasets were created: one for training and the other for testing various classifiers, considering five or seven postures. For both five and seven-class models, the best performance was achieved by the k-NN model in all evaluated metrics, and, as expected, the five-class model had higher classification accuracy (98.5%) than the seven-class (87.5%), as some positions considered by the latter (P7 and P8) have their CM in close proximity or overlapping the clusters of other positions (P1, P2, P5 and P6). In the end, the posture classifier chosen for the final prototype was the seven-class k-NN with 3000 neighbors, due to its higher classification accuracy for the P1 and P2 positions (commonly adopted by workers), and capability to identify a broader set of postures, making this prototype better suited to promote dynamic sitting.

Additionally, we were able to achieve these results using a much simpler approach both in terms of sensor and computational complexity compared to other SPMSs proposed in the literature, facilitating large scale deployment of the proposed system.

For ECG signal acquisition, results show that, while the signals acquired with the conductive nappa had decreased quality comparatively to the reference, they still maintained the morphology of the ECG signal for the most part, and, while certain noise sources could not be removed with filtering, using DBSCAN lead to an increase in R-peak detection accuracy that compensated the loss in performance.

In conclusion, the aforementioned experimental results indicate that the smart chair system proposed in this work is both feasible and well performing in its various monitoring modules. Despite the results obtained, future work will seek to further increase the performance of the various subsystems and facilitate the incorporation of the developed smart chair into the office environment. This includes: experimenting with other inexpensive sensors (e.g., infrared distance sensors) and adding new features to the dataset, to try and increase the posture classification accuracy and include more sitting positions; the development of a platform to wirelessly charge the smart chair, guaranteeing its complete independence from cabled connections to external devices; incorporating ECG leads-off detection at the hardware level, so as to avoid needless continuous ECG signal acquisition, which will occur as a natural extension of the user’s normal sitting activities; development of a web app to provide feedback to the user on their sitting posture and cardiovascular health, using the sensor data wirelessly transmitted by the SENSE to a real-time database, where they are processed on arrival and extracted metrics are stored; and usability experiments.

## Figures and Tables

**Table 1:** Posture monitoring systems proposed in the literature.

Papers	No. of Test Subjects	Sensor Type	Information Provided by Sensors	Sensor Location	Sitting Positions	Classification Method	Overall Accuracy
Martins et al. (2013) [21,22]	30	Piezoelectric gage pressure sensors	Pressure maps	4 inside the seat and 4 inside the backrest	5/8/11	ANN	98.1/93.4/81.8%
Zemp et al. (2015) [23]	20	Pressure sensor mat	Static pressure distribution parameters	On top of seat and backrest	3	-	-
Park et al. (2016) [24]	-	Fabric pressure and 4-level analog tilt sensors	Pressure readings and tilt level	Pressure sensors on the front and back of left and right seating pads, tilt sensors on the bottom of left and right seating pads	6	ET	-
Zemp et al. (2016) [25]	41	FSRs and IMU	Median of one-second duration of force data divided by the subject’s body weight (for each FSR) and of backrest angles	IMU attached to rear of backrest, 4 FSR fixed on backrest, and 3 FSR fixed on each armrest.	7	RF	90.9%
Huang et al. (2017) [18]	-	Piezo-resistive sensor array	Pressure map	On top of seat	8	ANN	92.2%
Otoda et al. (2018) [19]	20	3-axis accelerometers	Variation between tilt angle of the chair while sitting and tilt angle of initial state	6 on the back side of the seat and 2 on the back side of the backrest	18	RF	80.1%

**Table 2:** Posture monitoring systems proposed in the literature (continued).

Papers	No. of Test Subjects	Sensor Type	Information Provided	Sensor Location	Sitting Positions	Classification Method	Overall Accuracy
Roh et al. (2018) [17]	9	Load cell	Body weight ratio	4 on seat plate	6	SVM	97.2%
Kim et al. (2018) [26]	10	Pressure sensor mat	Heat map images of body pressure distribution	Inside chair seat cushion	5	CNN	95.3%
Ishac et al. (2018) [27]	20	Conductive fabric pressure sensing array	Deviation of sensor value errors from the validated calibration reference array	Cushion placed on top of backrest	11	ET	98.1%
Matuska et al. (2020) [28]	12	FSR	Average standard deviation from five measurements for bottom sensors	4 placed on seat, 2 placed on backrest	9	ET	-
Jeong et al. (2021) [20]	36	FSR and infrared reflective distance sensors	Normalized pressure and distance sensor measurements, along with pairwise differences of the normalized sensor measurements within each sensor type	6 FSR on seat cushion and 6 distance sensors on backrest	11	k-NN	92.0%
Wan et al. (2021) [29]	10	Pressure sensor array	Regional pressure values of the left hip, right hip and caudal vertebrae obtained from sitting pressure image	On top of seat	4	SVM	89.6%

**Table 3:** Comparative analysis of the heart rate and R-peak amplitude determined for the ECG time series obtained with the pre-gelled electrodes and with the conductive nappa, and SNR and cossine similarity between the scaled version of these signals.

Electrode Type	Outlier Detection	HR (bpm)	Amplitude (mV)	Performance (%)	Accuracy (%)	SNR (dB)	Cossine Similarity
Pre-gelled	**-**	82.04±4.66	297.62±16.69	-	-	-	-
Conductive Nappa	**-**	89.79±21.45	192.06±35.87	90.83±16.00	85.84±20.63	1.39±3.55	0.51±0.36
	DBSCAN	86.45±16.38	194.53±33.60	88.21±21.57	90.50±16.82		
	DMEAN	85.54±14.62	190.89±21.70	76.34±16.17	93.06±16.72		
	NCCC	83.82±11.02	195.86±29.92	69.14±30.78	94.41±16.89		

**Table 4:** Statistical analysis of the measured center of mass coordinates for each sitting position evaluated.

Sitting Position	Measured CM Statistics (cm)					
	${\mathrm{CM}}_{x}$			${\mathrm{CM}}_{y}$		
	μ±σ	Max	Min	μ±σ	Max	Min
P1	15.25±0.37	15.94	14.52	3.20±0.66	4.59	1.36
P2	15.20±0.36	16.08	14.27	5.99±0.68	8.66	4.67
P3	15.07±0.44	16.22	14.08	9.23±0.96	11.32	7.57
P4	15.45±0.49	16.77	14.26	2.26±2.10	6.39	0.00
P5	19.60±0.88	21.69	17.62	3.63±0.78	5.03	1.72
P6	10.83±0.99	12.80	7.96	3.92±0.79	6.05	2.05
P7	16.79±0.84	18.46	15.08	3.43±1.33	6.76	0.82
P8	13.63±0.79	14.78	11.51	3.32±1.46	6.46	0.38

**Table 5:** Optimal parameters of each classification model type trained.

No. of Classes	Classification Models	Optimal Hyperparameters
5	k-NN	Brute force algorithm Inverse distance function
	NC	Euclidean distance metric
	SVM	Linear kernel C = 0.001
	GMM	Tied covariance
7	k-NN	Brute force algorithm Inverse distance function
	NC	Cityblock distance metric
	SVM	Radial basis function kernel C = 0.1, γ = 0.5
	GMM	Full covariance

**Table 6:** Classification performance metrics for the models using the test dataset. The numbers in between parentheses indicate the number of neighbors used in the k-NN model.

No. of Classes	Classification Model	Classification Performance Metrics			
		Precision	Recall	F1-Score	Accuracy
5	k-NN (250)	0.980±0.023	0.980±0.019	0.980±0.021	0.980
	k-NN (500)	0.979±0.026	0.978±0.019	0.979±0.021	0.978
	k-NN (750)	0.980±0.023	0.980±0.019	0.980±0.019	0.980
	k-NN (1000)	0.985±0.017	0.985±0.018	0.985±0.015	0.985
	k-NN (2000)	0.983±0.020	0.983±0.019	0.983±0.016	0.983
	k-NN (3000)	0.981±0.024	0.980±0.018	0.980±0.019	0.980
	NC	0.979±0.027	0.978±0.019	0.978±0.021	0.978
	SVM	0.979±0.027	0.978±0.019	0.978±0.021	0.978
	GMM	0.975±0.030	0.974±0.021	0.974±0.024	0.974
7	k-NN (250)	0.871±0.115	0.869±0.120	0.868±0.113	0.869
	k-NN (500)	0.859±0.111	0.858±0.138	0.856±0.120	0.858
	k-NN (750)	0.862±0.110	0.861±0.140	0.858±0.121	0.861
	k-NN (1000)	0.868±0.104	0.867±0.141	0.864±0.118	0.867
	k-NN (2000)	0.877±0.092	0.876±0.151	0.872±0.117	0.876
	k-NN (3000)	0.876±0.092	0.875±0.158	0.869±0.121	0.875
	NC	0.855±0.122	0.849±0.181	0.843±0.147	0.849
	SVM	0.864±0.123	0.861±0.131	0.860±0.123	0.861
	GMM	0.797±0.171	0.754±0.234	0.749±0.173	0.754

**Table 7:** Predicted label relative frequency (%) in each transition period, for the k-NN model with 3000 neighbors.

Transition Period	Predicted Label Relative Frequency (%)						
	P1	P2	P3	P5	P6	P7	P8
P1-P2	22.86	64.05	7.60	0.00	0.00	0.36	5.13
P2-P3	2.99	36.10	59.30	0.00	0.00	0.00	1.61
P3-P5	3.07	9.50	26.97	53.43	1.40	3.40	2.23
P5-P6	0.33	3.64	2.98	25.04	63.37	1.66	2.98
P6-P7	33.70	11.74	5.03	3.50	17.03	19.90	9.10
P7-P8	13.36	17.58	8.91	0.99	1.53	12.75	44.88
Average	21.89	27.94	18.68	8.73	4.30	7.12	11.34

**Figure A1:**
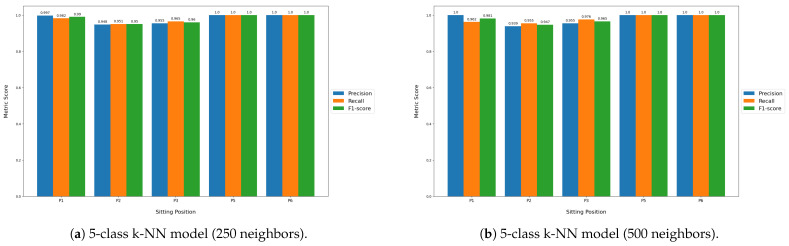
Label-wise precision, recall and F1-score values obtained for each classifier.

**Figure A2:**
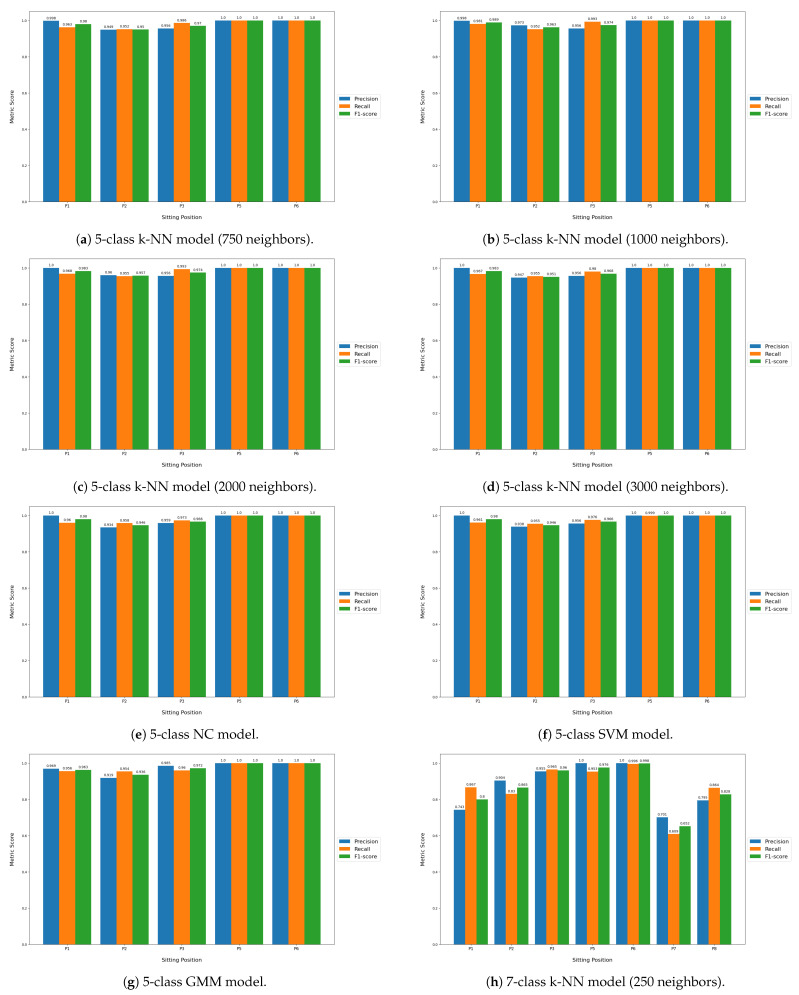
Label-wise precision, recall and F1-score values obtained for each classifier (cont.).

**Figure A3:**
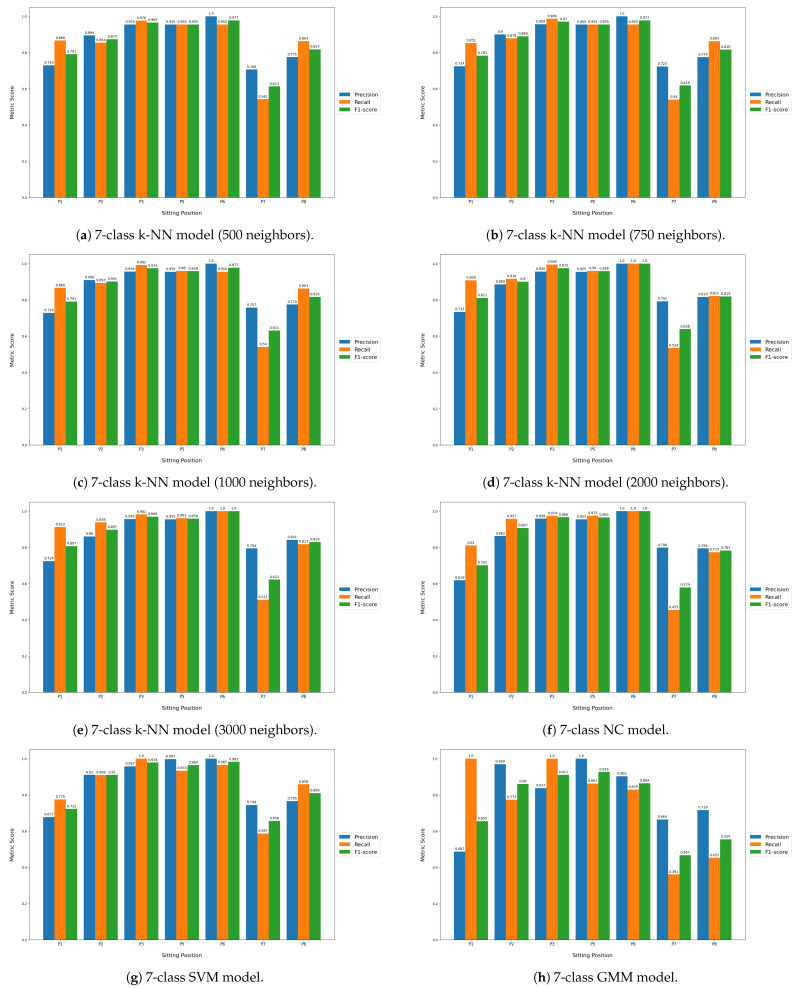
Label-wise precision, recall and F1-score values obtained for each classifier (2nd cont.).

**Figure A4:**
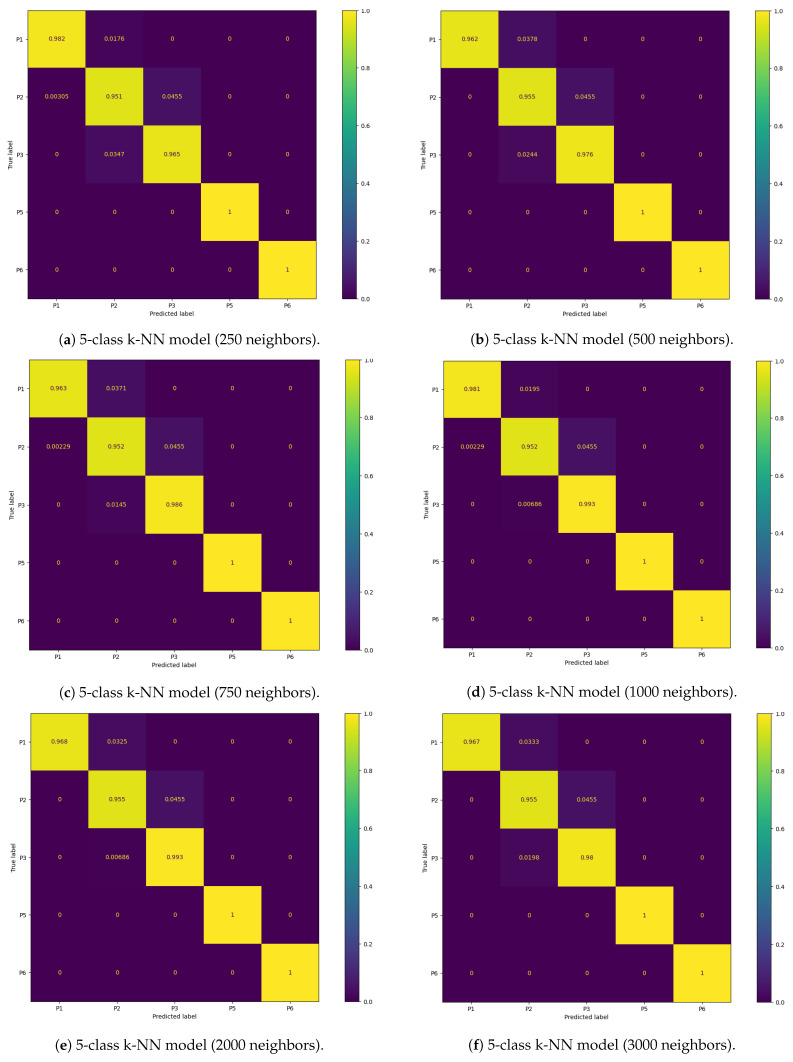
Confusion matrix of the classification results for each classifier.

**Figure A5:**
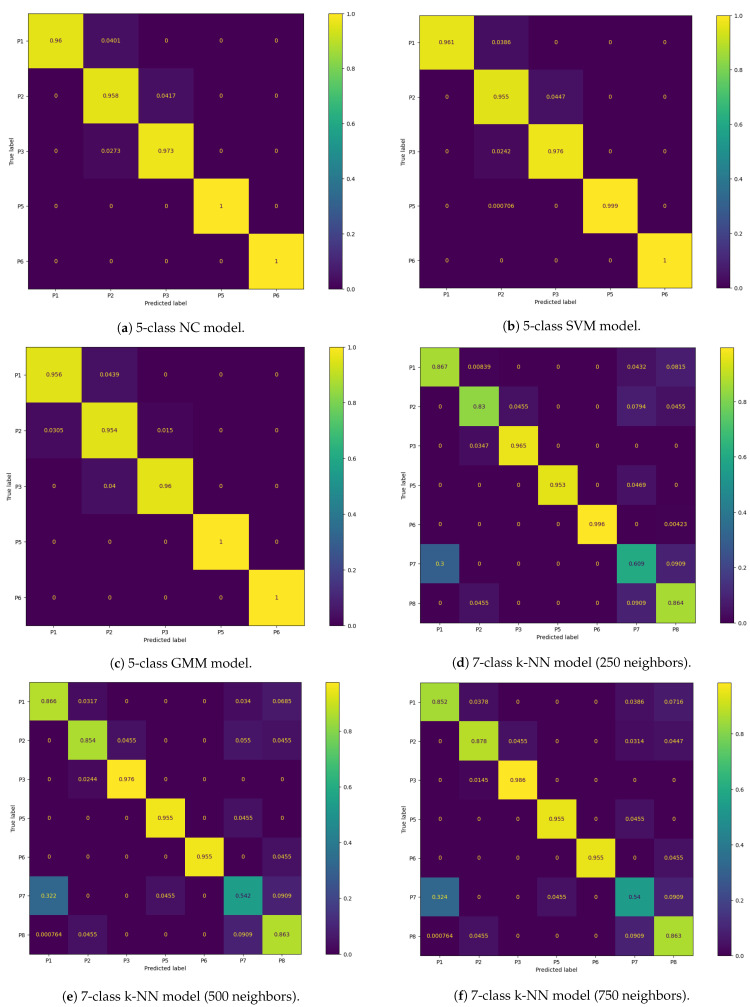
Confusion matrix of the classification results for each classifier (cont.).

**Figure A6:**
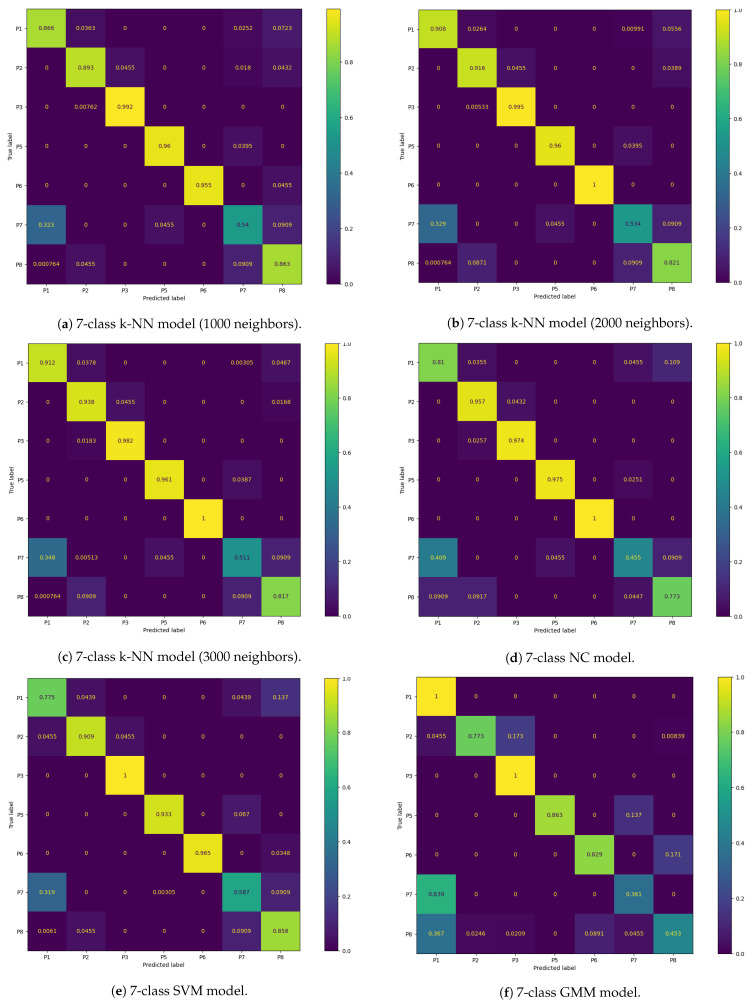
Confusion matrix of the classification results for each classifier (2nd cont.).

**Figure A7:**
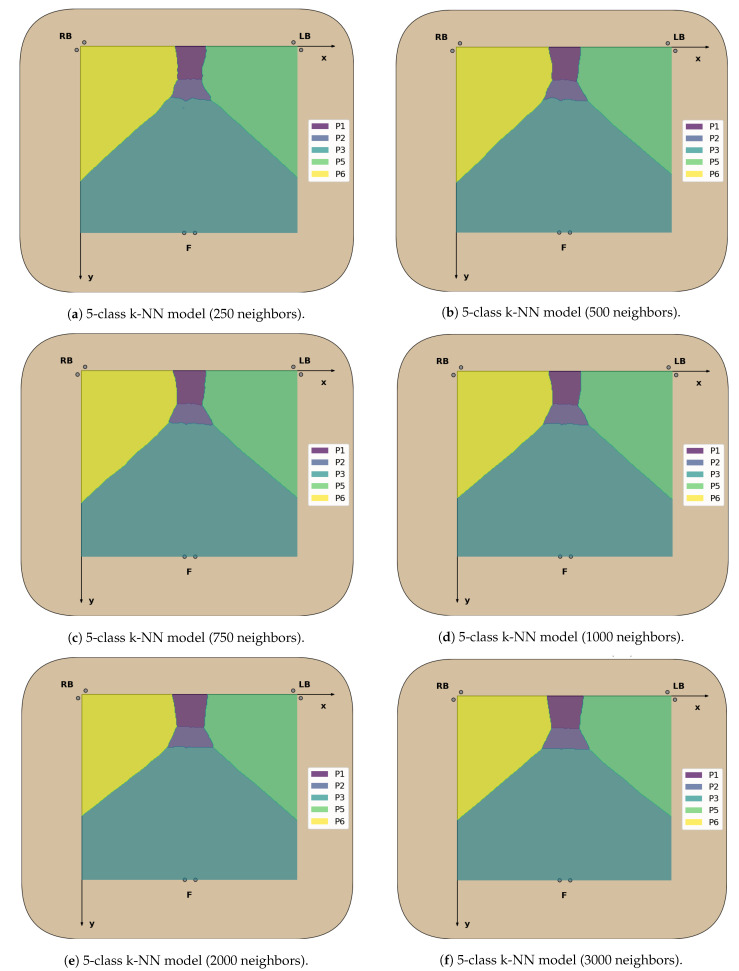
Decision boundaries in the 2D chair seat plane for each classifier trained.

**Figure A8:**
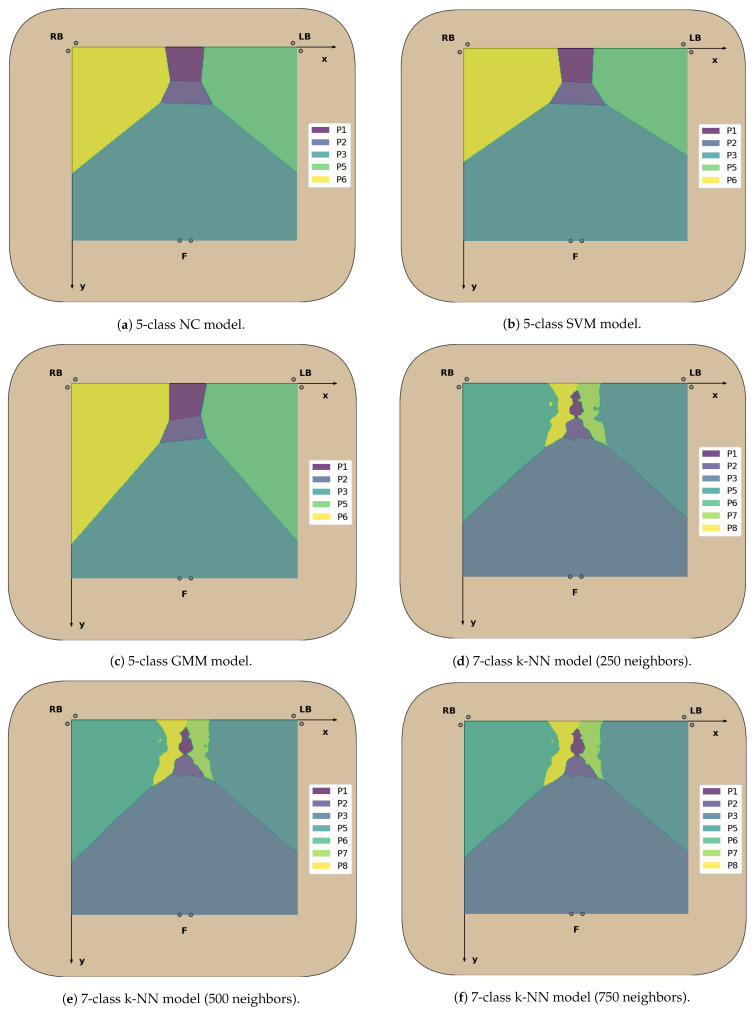
Decision boundaries in the 2D chair seat plane for each classifier trained (cont.).

**Figure A9:**
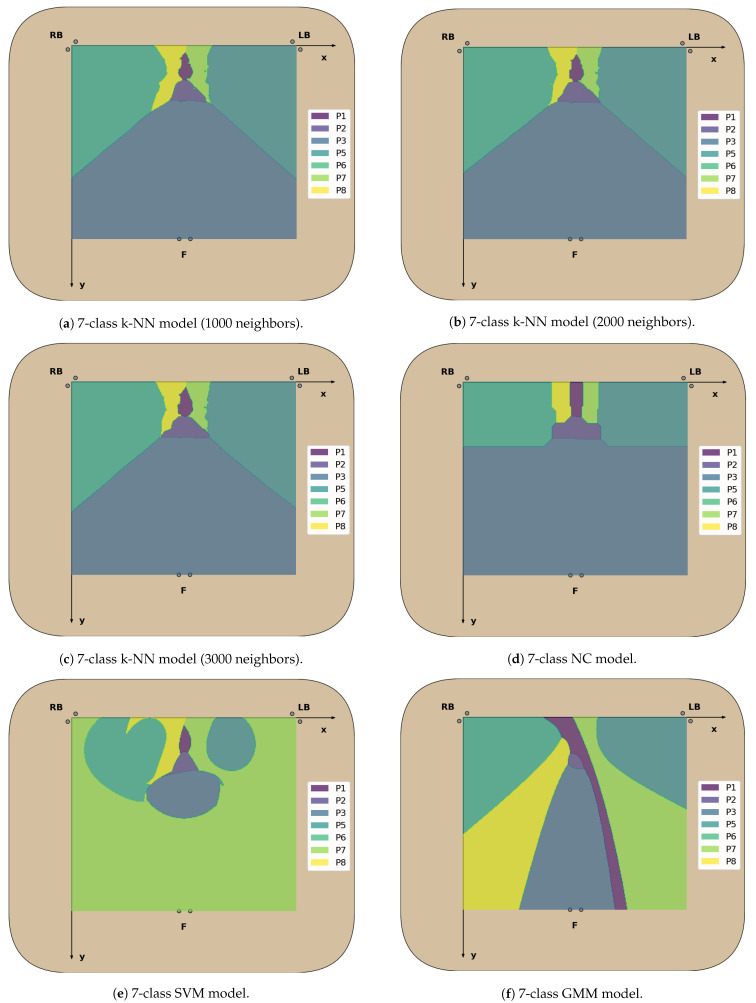
Decision boundaries in the 2D chair seat plane for each classifier trained (2nd cont.).

## Data Availability

The data presented in this study are available upon reasonable request made to the corresponding author. The data are not publicly available due to privacy restrictions.

## Abbreviations

The following abbreviations are used in this manuscript:

ADC	Analog-to-Digital Converter
ANN	Artificial Neural Network
CM	Center of Mass
CNN	Convolutional Neural Network
CV	Cross Validation
CVDs	Cardiovascular Diseases
ECG	Electrocardiography
ET	Empirical Threshold
FIR	Finite Impulse Response
FSR	Force Sensitive Resistor
GMM	Gaussian Mixture Model
k-NN	k-Nearest Neighbors
MSDs	Musculoskeletal Disorders
NC	Nearest Centroid
OSH	Occupational Safety and Health
RF	Random Forest
RLD	Right Leg Drive
SPMSs	Sitting Posture Monitoring Systems
SVM	Support Vector Machine

## Appendix A

**Table A1:** Hyperparameter spaced used for each classification model.

Classification Model	Hyperparameter Space
**k-NN**	**No. of neighbors *:** {250, 500, 750, 1000, 2000, 3000} **Weight function:** {uniform, inverse of the distance} **Algorithm:** {Brute force, K-dimensional tree, Ball tree} **Leaf size:** {10, 20, 30, 40, 50}
**CN**	**Distance metric:** {Cityblock, Cosine, Euclidean, L1, L2, Manhattan}
**SVM**	**Kernel:** {Radial basis function, Linear, Polynomial, Sigmoid} **C:** {0.0001, 0.001, 0.01, 0.1} **Gamma:** {$1/(n_{features)}$, $1/(n_{features}\times Var\left[X\right])$} **Degree:** {3, 4, 5}
**GMM**	**Covariance type:** {Full, Tied, Diagonal, Spherical}

* Manually fixed hyperparameter.
